# Bacterial Respiration Used as a Proxy to Evaluate the Bacterial Load in Cooling Towers

**DOI:** 10.3390/s20216398

**Published:** 2020-11-09

**Authors:** Stepan Toman, Bruno Kiilerich, Ian P.G. Marshall, Klaus Koren

**Affiliations:** 1 Centre for Water Technology, Department of Biology, Section for Microbiology, Aarhus University, Ny Munkegade 114, 8000 Aarhus, Denmark; stepatoma@gmail.com; 2 Grundfos Holding A/S, Poul Due Jensens Vej 7, 8850 Bjerringbro, Denmark; bkiilerich@grundfos.com; 3 Center for Electromicrobiology, Department of Biology, Section for Microbiology, Aarhus University, Ny Munkegade 114, 8000 Aarhus, Denmark; ianpgm@bio.au.dk

**Keywords:** oxygen, optode, cooling tower, industrial monitoring, bacterial analysis

## Abstract

Evaporative cooling towers to dissipate excess process heat are essential installations in a variety of industries. The constantly moist environment enables substantial microbial growth, causing both operative challenges (e.g., biocorrosion) as well as health risks due to the potential aerosolization of pathogens. Currently, bacterial levels are monitored using rather slow and infrequent sampling and cultivation approaches. In this study, we describe the use of metabolic activity, namely oxygen respiration, as an alternative measure of bacterial load within cooling tower waters. This method is based on optical oxygen sensors that enable an accurate measurement of oxygen consumption within a closed volume. We show that oxygen consumption correlates with currently used cultivation-based methods (R^2^ = 0.9648). The limit of detection (LOD) for respiration-based bacterial quantification was found to be equal to 1.16 × 10^4^ colony forming units (CFU)/mL. Contrary to the cultivation method, this approach enables faster assessment of the bacterial load with a measurement time of just 30 min compared to 48 h needed for cultivation-based measurements. Furthermore, this approach has the potential to be integrated and automated. Therefore, this method could contribute to more robust and reliable monitoring of bacterial contamination within cooling towers and subsequently increase operational stability and reduce health risks.

## 1. Introduction

**Figure 1:**
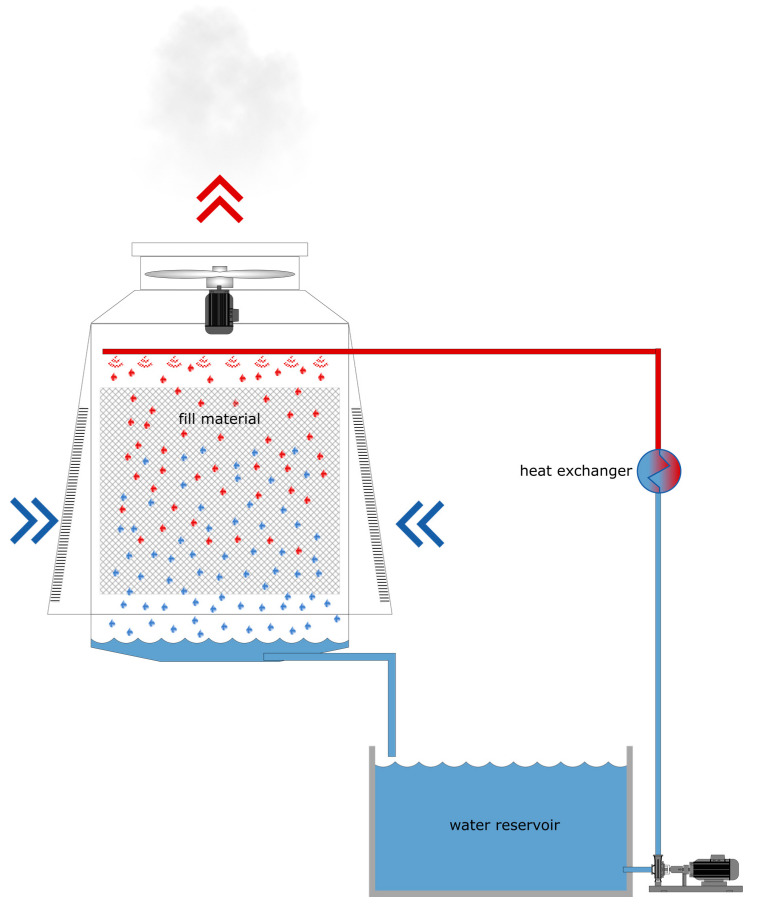
Sketch of an evaporative cooling tower with a water reservoir, fill material and heat exchanger. Microbial communities can thrive within the humid environment, both in planktonic form as well as in biofilms on all surfaces.

Evaporative cooling towers are essential systems used in industry to dissipate excess process heat. Several types of evaporative cooling towers exist and all of them contain a reservoir of cooling water. To dissipate the process heat, the cold water from the reservoir is pumped over a heat exchanger where the heat is transferred to the water. From there, the warm water is transferred to the top of the cooling tower and distributed by spray nozzles over a fill material (Figure 1). As water runs down through the fill material, some of it evaporates. This evaporation is further enhanced if the fans of the cooling tower are also in action, inducing an upward flow of air in the tower. Evaporation extracts the energy from the water and cools it. The water that does not evaporate flows back to the water reservoir with a concentrated amount of minerals [1,2]. 

The humid environment within the cooling tower favors the growth of diverse planktonic and sessile microbial communities [3]. The sessile microbial growth reduces the efficiency of the cooling tower as biofilms develop on surfaces, including those of the heat exchanger [4], where they reduce heat transfer significantly [5]. Furthermore, biocorrosion [6,7] also poses a risk to the structural parts of cooling towers [5,8]. In addition, pathogenic microbes, which can be part of the community within cooling towers pose a substantial health risk, as they can be aerosolized and inhaled by people in the vicinity of the towers [9]. 

While both bacteria and protists can be found in cooling towers, bacteria are more abundant and critical [10]. Human pathogens [11] like certain *Legionella* spp. pose a great risk, and extensive growth of such pathogens must be avoided [12]. *Legionella* spp. are Gram-negative aerobic bacteria that can be found in natural water reservoirs [13]. Some *Legionella* spp. can be harmful to humans when inhaled, causing a pneumonia-like disease (Legionnaires’ disease) [9]. The discovery of this disease dates back to an outbreak in 1976, in which 29 of 182 cases were fatal [14]. Several outbreaks of Legionnaires’ disease connected to cooling towers have been reported [15,16,17,18] with an approximate case fatality rate of 6% [19]. The European Technical Guidelines for the Prevention, Control and Investigation of Infections Caused by *Legionella* species prescribes zero tolerance of *Legionella* presence in cooling towers. When *Legionella* in excess of 10,000 colony forming units (CFUs) per liter are detected in a cooling tower, immediate shut down is required and particular actions must be taken, including immediate resampling to confirm earlier measurements [20]. Given the negative impacts that bacterial growth within cooling towers has on the economy and safety of the process, it is essential to monitor bacterial concentrations in cooling towers at all times, and to take appropriate disinfection measures by adding biocides to the cooling water. 

Currently, cooling towers are surveyed with respect to bacterial load using grab sampling and subsequent cultivation approaches. This means that small volumes of water are sampled and the bacterial load is quantified by determining the number of CFUs. However, all cultivation-based methods have a so-called cultivation bias [21]. This means that not all microorganisms that are present in a liquid sample will form colonies on a given cultivation medium. Although the often used dip-slide cultivation method is a rather fast sampling and preparation method, cultivation-based methods are generally slow, as it takes 1–2 days to produce the actual result after sampling. In addition to the length of time between sampling and results, grab sample-based analysis also requires samples to be taken at regular intervals by trained personnel. As this is expensive and laborious, cooling towers are often only surveyed on a monthly basis. Given the fact that bacterial populations can grow rapidly if the conditions allow it, such sparse sampling intervals are not sufficient. Thus, current practices hardly give an accurate picture of the actual loads in cooling waters. Furthermore, measurement of the bacterial load is crucial for regulating the addition of biocides to the cooling water, in order to keep the bacterial load under control while avoiding excessive use of chemicals. 

In this study, we present a novel approach that uses bacterial respiration as a proxy for real-time evaluation of the microbial load in the cooling water of evaporative cooling towers. To the best of our knowledge, such an approach has not been demonstrated before. An optical sensor is used to measure the oxygen content of cooling water in a closed container. The deduced respiration rate is correlated with the number of microorganisms, and thus provides a possible real-time determination of bacterial load in the cooling tower. This can potentially be used to control the addition of biocides to the cooling tower reservoir more precisely than current practices. Ultimately, real-time monitoring of cooling towers can decrease health and environmental risks associated with bacterial growth within such systems. 

## 2. Materials and Methods

### 2.1. Sampling

Samples of cooling water and biofilms were collected from a cooling tower at the premises of Grundfos A/S (Bjerringbro, Denmark) multiple times throughout the second half of 2019. All samples were taken within one hour to minimize sample content dissimilarity. For flow cytometry measurements, the liquid samples were stored in 5 L batches at 4 °C and biofilm samples were stored in Eppendorf tubes at −80 °C until analysis was carried out (within 24 h). For oxygen respiration measurements, the liquid samples were kept at room temperature before the start of the experiment (for less than 3 h). A flow chart showing the workflow from sampling to the respective analyses is provided in the supporting information (Figure S1). 

### 2.2. Quantitative Analyses

#### 2.2.1. Cultivation-Based Methods

The reservoir water samples were analyzed quantitatively using both regular plates as well as dip slides (Cult-Dip, Merck KGaA, Germany) to determine the presence of viable cells. First, a 1 mL sample of well-mixed reservoir water was inoculated on Plate Count Agar (PCA) (Bio-Rad, USA), a nonselective medium. The PCA plates were incubated at 28 °C for 48 h and colonies were subsequently counted. In accordance with the manufacturer’s guidelines, the dip slides were immersed into the properly mixed reservoir water samples and incubated at 28 °C for 48 h. The number of viable cells was quantified by comparing growth intensity to the scale provided with the test kit.

#### 2.2.2. Flow Cytometry

Water and biofilm samples were analyzed by flow cytometry (NovoCyte Quanteon, ACEA Biosciences, USA) at the Fluorescence Activated Cell Sorting Core Facility (FACS) at Aarhus University. Liquid samples were analyzed for viable and nonviable cells using two different staining solutions. The SYTO™ 9 Green Fluorescent Nucleic Acid Stain (Thermo Fisher Scientific, USA) was chosen for visualizing viable and nonviable cells, using an excitation wavelength of 488 nm with collection of fluorescence emission at 530/30. Next, propidium iodide (Thermo Fisher Scientific, USA) was selected for the detection of viable cells with an excitation laser at 561 nm and bandpass emission filter at 615/20. Each round of sample detection was performed for 9.3 min with a sample speed of 9 µL/min. The data from the flow cytometry analysis were processed using NovoExpress software (ACEA Biosciences, San Diego, CA, USA).

#### 2.2.3. Oxygen Respiration Measurements 

**Figure 2:**
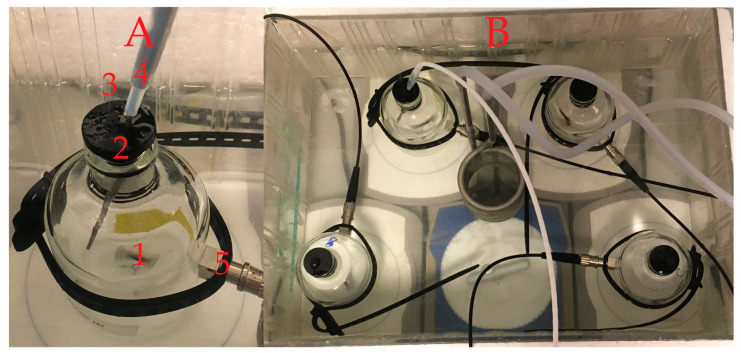
(**A**): Picture of the measurement vial with the glass magnet (1), rubber stopper (2), thin glass capillary (3), temperature probe (4) and O_2_ sensor spot with the respective connector (5). (**B**): Three samples and one control measured at the same time within a water bath kept at 22 °C.

Bacterial oxygen consumption was measured in the liquid samples using a FireStingO2 Optical Oxygen Meter in combination with an OXSP5 sensor spot (both from PyroScience GmbH, Aachen, Germany). The meter features a temperature channel, used for temperature compensation, and four oxygen channels. Sensor spots were glued inside later sterilized (acid and base washed) vials (116 mL). Each vial was equipped with a sterilized glass magnet (1) and filled with the liquid samples. The vial was properly sealed with a rubber stopper (made from butyl rubber) to prevent oxygen exchange (2). To maintain stable pressure, a thin glass capillary was inserted through the rubber stopper (3). The temperature was compensated using a temperature probe (4) (see Figure 2). The FireStingO2 Optical Oxygen Meter was connected to each of the vials using a one-meter long optical fiber (SPFIB-BARE) and a lens spot adapter (SPADBAS), both from PyroScience GmbH, Aachen, Germany (5). A Pyro Oxygen Logger (PyroScience GmbH, Aachen, Germany) was used for data collection and processing. A table summarizing the respective equipment needed to set up such a respiration measurement is provided in the supporting information (Table S1). Two-point calibration of the sensor was performed prior to each set of measurements using 100% air-saturated water and O_2_-free water as described by the provider. Measurements were performed simultaneously on three liquid samples from the cooling tower reservoir and one control sample containing demineralized water (ddH2O) at 22 °C (20 °C for the measurements shown in Figure 4). All samples and control samples (66 mL) were supplemented with 25 mL 1 M glucose and 25 mL LB broth medium (both from Sigma-Aldrich and autoclaved prior to addition). 

#### 2.2.4. Bacterial Community Analysis

For closer examination of the cooling tower samples, taxonomic identification by 16S rRNA gene sequencing was performed. Both liquid and biofilm samples were analyzed. A liquid sample of 100 mL was filtered using a 0.2 µm filter. Additionally, a number of colonies from the PCA plates were picked for sequencing. DNA was extracted from the samples using an MP Biomedicals™ FastDNA™ SPIN Kit for Soil (MP Biomedicals, Eschwege, Germany). Negative control extractions were made without the addition of sample material. Several 16S rRNA gene amplicon libraries were prepared according to Illumina’s 16S Metagenomic Sequencing Library Preparation guide, with slight modifications, using Bac341F and Bac805R primers to amplify variable regions V3 and V4 [22]. Sequencing was carried out using an Illumina MiSeq.

Primers were trimmed from the ends of the forward and reverse reads using cutadapt, version 1.15 [23]. Sequences were then processed into amplicon sequence variants (ASVs), chimeras removed, and classified using DADA2, version 1.12.1 [24]. The SILVA database, version 132, was used for classification [25]. Principal coordinates analysis was carried out on Bray–Curtis distances between relative abundances of ASVs using the phyloseq package, version 1.32.0 [26]. A heatmap was drawn using the ampvis2 package, version 2.6.4 [27]. Similarities between the cultivated and reservoir communities were measured using the ANOSIM function with Bray–Curtis distances in the vegan package, version 2.5.6.

## 3. Results and Discussion

### 3.1. Cultivation-Based Methods and Community Analysis

The objective of this study is to use metabolic activity, in this case, oxygen respiration, as a proxy for the bacterial load within the water of cooling towers. As cooling towers are essential units in multiple processes, maintaining a low and pathogen-free bacterial load is essential. Firstly, cultivation-based methods representing the current standard were evaluated against flow cytometry to measure the extent to which these cultivation methods are able to quantify the total bacterial load in the cooling water. Reservoir water samples were analyzed using two different total viable count methods in order to explore the potential differences between the methods. The dip slides determined the total viable cell count of 1.00 × 10^5^ CFU/mL. However, dip slides are a semiquantitative method for which precision is limited to five intervals according to the measuring scale of the manufacture. The standard plate count method using PCA media plates resulted in a lower CFU/mL value (5.80 × 10^4^ CFU/mL) compared to the method using dip slides.

As an alternative to counting colony forming units according to growth, bacterial cells can be counted using a flow cytometer after being appropriately stained with fluorescent dyes [28]. This method resulted in the highest number of total cells counted within the same reservoir water. Flow cytometry resulted in an average of 2.01 × 10^6^ live cells/mL. There is a difference of one to two orders of magnitude compared to cultivation methods (5.80 × 10^4^ live cells/mL using the plate count method and 1.00 × 10^5^ live cells/mL using the dip slides method), which is related to the cultivation bias. It is well known that only a fraction of the bacteria within a sample can actually grow on the provided medium when using agar plates [29,30]. Even though flow cytometry provides a more complete representation of the bacterial load, the plate count method is the current standard due to its simplicity and the possibility of performing it in almost any laboratory. As mentioned previously, flow cytometry requires staining of the sample and the use of a rather expensive instrument, which hinders widespread application of this methodology, particularly with regard to online measurements. 

**Figure 3:**
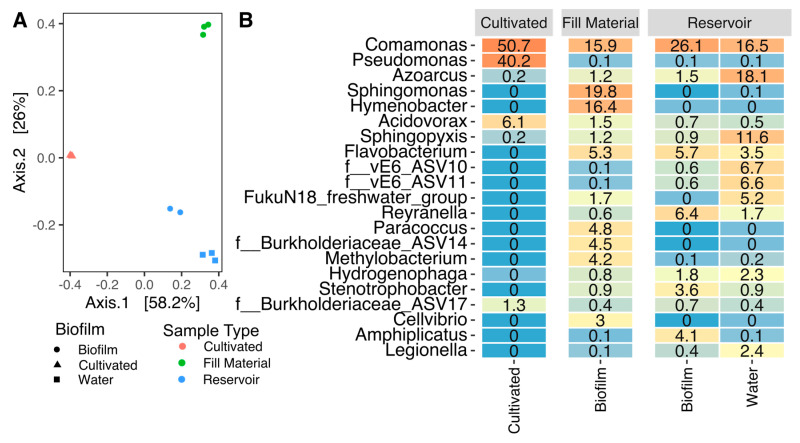
(**A**): Principal Coordinates Analysis based on Bray–Curtis distances between ASV relative abundances. The two most significant principal components are shown, axes annotated with percentage of variation explained. (**B**): Heatmap showing mean percentage abundances for all sample types for the 21 most abundant genera in the dataset. Rows labeled with names ending in “_ASVXX” show ASVs with undefined genera (only defined up to the family level) which are nonetheless more abundant than all genera in the top 21. vE6 is an uncultured family-level group within the Chlamydiales.

The bacterial community analysis showed a much greater difference in community composition between cultivated samples and directly sequenced samples than between replicates, thus demonstrating that cultivation produces significant biases in the observed bacterial community profile of this system (Figure 3A) and reinforcing the numerical discrepancy observed between CFU counts and flow cytometry measurements. This visual impression is supported by the ANOSIM statistical test which shows a significant difference between cultivated and directly sequenced bacterial communities (ANOSIM R = 0.71, *p* = 0.002). This is also evident from the abundances of different genera in the system (Figure 3B): for example, while 40% of the 16S rRNA gene sequences from cultivated bacteria were *Pseudomonas*, this genus only constituted 0.1% of the reservoir sequences. Furthermore, a whole range of bacterial genera that were present in the reservoir were not cultivated, including important potential pathogens such as *Legionella*, comprising up to 2.4% of the sequencing reads (Figure 3A). Samples from the reservoir biofilm and water cluster together as shown in Figure 3A, demonstrating that the bacterial communities in the reservoir water and in the biofilm sample from the reservoir were rather similar. In contrast, the biofilm sample collected from the fill material showed a somewhat different community composition. This is largely in agreement with a previous study that showed that biofilm communities differ with respect to the local temperature [31]. In this case, the fill material with its attached biofilm was exposed to warm water while the reservoir temperature was lower and, on average, more constant. In addition to this, seasonal fluctuations can and do occur, as described elsewhere [10]. In conclusion, monitoring bacteria in water is likely to be an effective and the most relevant way of monitoring, as it is these bacteria that will be aerosolized, and therefore, potentially cause a health risk. At the same time, water monitoring does not necessarily provide information on the bacterial communities present within the biofilms found within a system.

### 3.2. Oxygen Respiration Measurements

While both cultivation and flow cytometry count numbers of cells, metabolic measurements use a different approach. By measuring the consumption or production of a metabolite, these types of measurements link metabolic activity to abundance. Bacterial respiration is an established method in monitoring food spoilage [31,32,33] as well as within environmental analysis [34,35,36], but has so far not been intensively studied as an option within cooling tower monitoring. In situ measurements showed that the reservoir water was fully air-saturated, implying that measuring oxygen consumption, and therefore aerobic metabolism, should be a good proxy for the total bacterial load. 

**Figure 4:**
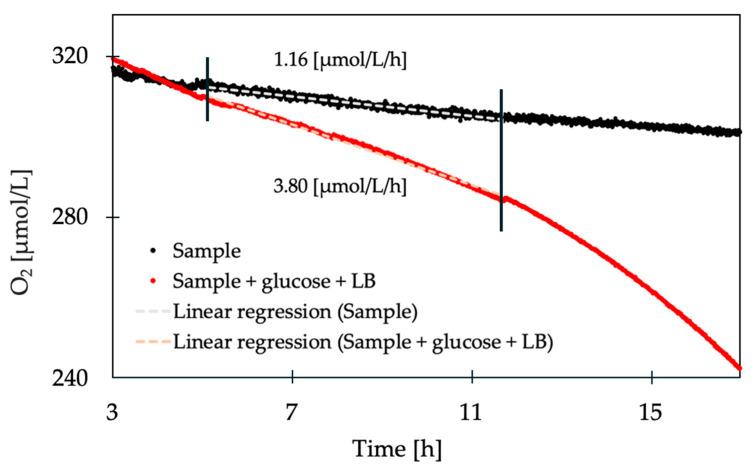
Comparison of the decrease in O_2_ concentration over time of a pure sample compared to a sample with added glucose and LB medium. The respective respiration rates were determined via linear regression and are displayed within the graph.

As expected, the reservoir water samples showed a respiration rate during incubation (Figure 4). The initial measurements showed highly variable rates and lacked reproducibility. However, when supplementing the sample (66 mL) with 25 mL 1M glucose and a 25 mL LB medium, the respiration rate increased by around three times (from 1.16 to 3.80 µmol/L/h). This implies that the respiration rate within cooling water is limited and influenced by the available nutrients, as has also been shown in a previous study [37]. If the reservoir water samples are not supplemented with micro- and macro- nutrients, the measurements would be biased toward the specific water matrix present at the time point of sampling. The addition of micro- and macro- nutrients therefore ensures the respiration rate depends solely on the bacterial load and not on a combination of nutrient availability and bacterial concentration. In addition, the bacterial growth phase has an impact on the respiration rate [38]. This can be observed in Figure 4, in which the oxygen consumption rate increases with time, indicating a transition in the growth phase. 

**Figure 5:**
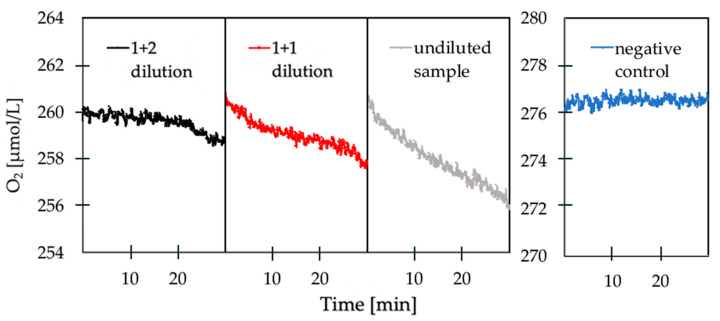
Examples of measured oxygen respiration of an undiluted sample, a 1 + 1 diluted sample and a 1 + 2 diluted sample compared to the negative control.

Once the dependency on nutrient availability was removed, the rate of oxygen consumption could be directly correlated with the bacterial load, and showed consistent results between sampling times. Figure 5 demonstrates the change in oxygen consumption between the undiluted sample, a 1 + 1 (sample + ddH2O) dilution and a 1+2 dilution compared to the negative control. There is a clear trend showing that an increasing dilution of the reservoir water samples is accompanied by a decreasing oxygen consumption rate. Correspondingly, Figure 5 also indicates that the dilution with ratio 1 + 2 results in a low oxygen consumption due to the low number of bacteria in the sample after dilution.

**Figure 6:**
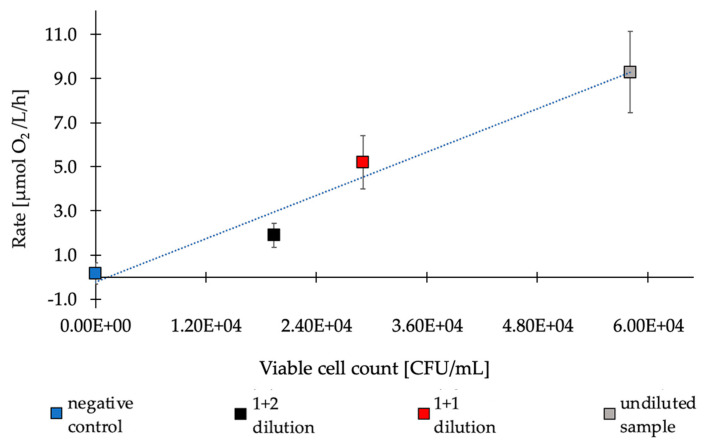
Correlation between the numbers of colony forming units (CFUs) in samples measured by plate counts and the oxygen consumption rates measured by the optode system. Data points represent means with the respective standard deviations for each sample (n = 5 for the undiluted sample, 2 for 1 + 1 and 1 + 2 dilutions and 3 for the negative control). The linear regression shown by the blue dotted line has an R^2^ = 0.9648.

The same dilutions of the reservoir water sample(s) were analyzed using both plate counts (CFU/mL) and the oxygen respiration method (µmol O_2_/L/h) to identify the correlation between the number of cultivable bacteria present in the reservoir water sample and oxygen consumption (Figure 6). We found a linear correlation between the two measurements with an R^2^ value of 0.9648. Using a 0.05 significance level, the two diluted samples differed significantly from the undiluted sample (p-value 0.0064 and 0.0003) but not from each other (*p*-value 0.0716). This is likely due to the rather low respiration rate of the most diluted sample. At the current stage, we determined the limit of detection (LOD) for our respiration measurement to be equal to 1.16 × 10^4^ CFU/mL. At rates of around 1 µmol O_2_/L/h, the resolution of the sensor (specified at around 0.6 µmol/L at air saturation by the manufacturer) can become a limiting factor. This could be overcome in the future by using sensors with oxygen indicators that are more sensitive. For example, optode systems have already been used successfully to measure in the nM range [39,40]. Furthermore, it is possible to tune the sensitivity of the sensor by other means, such as using a different matrix polymer as shown in a previous study [41]. Nevertheless, as oxygen consumption can clearly be correlated with bacterial load; this makes it possible to assess the amount of bacteria within a sample with a measurement time of around 30 min [37]. This is a significant reduction of the time between sampling and analysis, compared to the current 48 h taken by cultivation-based methods. 

## 4. Conclusions and Perspective 

Cooling towers are essential units within industrial production that need to be monitored with regard to their bacterial load. The current standard practice is to take grab samples and use slow cultivation-based methods for surveillance (48 h from sampling until result). Besides being slow, cultivation-based methods do not account for the bacterial diversity within a sample, since only a small fraction of the bacteria present can be cultivated, as we showed using 16S rRNA gene-based community analysis and flow cytometry. In this study, we presented an alternative approach using metabolic activity as a proxy for the bacterial load. By adding sufficient nutrients, it was possible to correlate the oxygen consumption rate with the bacterial load, resulting in a significantly shortened time of analysis (30 min compared to 48 h). This was demonstrated by measuring a real sample from a cooling tower using both approaches. Assessing multiple cooling towers in different locations would be the next step in order to account for variations in climate, season and cooling tower design. At the current proof-of-concept stage, those types of variations have not yet been accounted for. In order to establish a global correlation between respiration rates and CFU counts, such an extended survey would definitely be needed. Although we have only demonstrated the principle of the method as an offline measurement in this study, it has the potential for online integration. This could be a major step toward real-time bacterial monitoring and control of biocide dosage within cooling towers, a step needed to protect both workers and the environment, and to ensure uninterrupted operation.

## Supplementary Materials

The following are available online at https://www.mdpi.com/1424-8220/20/21/6398/s1, Table S1: Oxygen Respiration measurement setup, Figure S1: Flowchart visualising the respective analyses (in yellow) that were carried out for the sample obtained.
