# Supplementary material for: Bacterial Respiration Used as a Proxy to Evaluate the Bacterial Load in Cooling Towers
Source: Sensors (Basel). 2020 Nov 9;20(21):6398. doi: 10.3390/s20216398 (PMC7665125; doi:10.3390/s20216398)
Supplement: Supplementary file 1 [file sensors-20-06398-s001.pdf]

Table S1. Oxygen Respiration measurement setup.

OXSP5 sensor spot	PyroScience GmbH, Aachen, Germany
FireStingO2 Optical Oxygen Meter	PyroScience GmbH, Aachen, Germany
Temperature probe	PyroScience GmbH, Aachen, Germany
One-meter long optical fiber (SPFIB-BARE)	PyroScience GmbH, Aachen, Germany
Lens spot adapter (SPADBAS)	PyroScience GmbH, Aachen, Germany
Pyro Oxygen Logger software	PyroScience GmbH, Aachen, Germany
Water vials (116 mL)	
Glass magnet	Home made
Rubber stopper from butyl rubber	
Glass capillary	

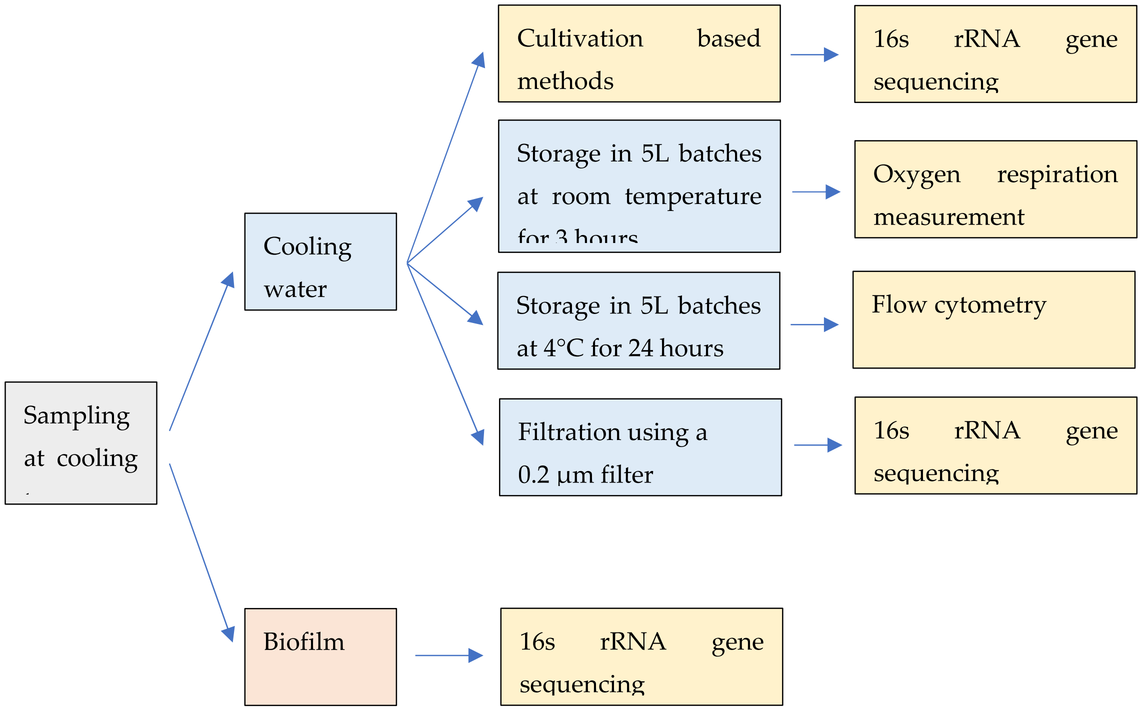

Figure S1. Flowchart visualising the respective analyses (in yellow) that were carried out for the sample obtained.
